# Suppressive response of confections containing the extractive from leaves of *Morus Alba *on postprandial blood glucose and insulin in healthy human subjects

**DOI:** 10.1186/1743-7075-6-29

**Published:** 2009-07-14

**Authors:** Mariko Nakamura, Sadako Nakamura, Tsuneyuki Oku

**Affiliations:** 1 Graduate School of Human Health Science, Siebold University of Nagasaki, 1-1-1 Manabino, Nagayo, Nagasaki, 851-2195, Japan

## Abstract

**Background:**

The first aim of this study was to clarify the effective ratio of extractive from leaves of *Morus Alba *(ELM) to sucrose so as to apply this knowledge to the preparation of confections that could effectively suppress the elevation of postprandial blood glucose and insulin. The second aim was to identify the efficacy of confections prepared with the optimally effective ratio determined from the first study, using healthy human subjects.

**Methods:**

Ten healthy females (22.3 years, BMI 21.4 kg/m^2^) participated in this within-subject, repeated measures study. For the first aim of this study, the test solutions containing 30 g of sucrose and 1.2 or 3.0 g of ELM were repeatedly and randomly given to each subject. To identify the practically suppressive effects on postprandial blood glucose and insulin, some confections with added ELM were prepared as follows: Mizu-yokan, 30 g of sucrose with the addition of 1.5 or 3.0 g ELM; Daifuku-mochi, 9.0 g of starch in addition to 30 g of sucrose and 1.5 or 3.0 g ELM; Chiffon-cake, 24 g of sucrose, starch, and 3.0 or 6.0 g of ELM, and were ingested by each subject. Blood and end-expiration were collected at selected periods after test food ingestion.

**Results:**

When 30 g of sucrose with 1.2 or 3.0 g of ELM were ingested by subjects, the elevations of postprandial blood glucose and insulin were effectively suppressed (*p *< 0.01), and the most effective ratio of ELM to sucrose was evaluated to be 1:10. AUC (area under the curve) of breath hydrogen excretion for 6 h after the ingestion of an added 3 g of ELM significantly increased (*p *< 0.01). When AUCs-3h of incremental blood glucose of confections without ELM was 100, that of Mizu-yokan and Daifuku-mochi with the ratio (1:10) of ELM to sucrose was decreased to 53.4 and 58.2, respectively. Chiffon-cake added one-fourth ELM was 29.0.

**Conclusion:**

ELM-containing confections for which the ratio of ELM and sucrose is one-tenth effectively suppress the postprandial blood glucose and insulin by inhibiting the intestinal sucrase, thus creating a prebiotic effect. The development of confections with ELM can therefore contribute to the prevention and the quality of life for prediabetic and diabetic patients.

## Background

It has recently become important for not only adults but also children to prevent metabolic syndrome, particularly when prevention of hyperglycemia and hyperlipidemia is also an issue [1-3]. In order to decrease the incidence and prevalence of life-style related diseases, it is necessary to develop processed foods that do not stimulate the elevation of postprandial blood glucose and secretion of insulin and that have lower energy. Materials that delay or inhibit digestion or absorption of carbohydrates could therefore be helpful in developing foods with health benefits. Soluble dietary fibers, alpha-glucosidase inhibitors, and polyphenols are known to be food ingredients with beneficial health effects. Some of them have already been used for foods with health claims regulated by the Health Promotion Act in Japan [4,5].

*Morus alba *contains 1-deoxynojirimycin (DNJ) and some of its derivatives, which are well known as an alpha-glucosidase inhibitors [6], and acarbose, boglibose and miglitol have already been used as medicines for the treatment of diabetes mellitus [7-13]. We have previously clarified that the extractive from the leaves of *Morus Alba *(ELM) competitively inhibits the activity of sucrase, maltase, and isomaltase using human and rat intestinal homogenates and significantly suppresses the increment of blood glucose levels when ELM was administered with sucrose in *in vivo *experiments using rats [14]. In addition, it has been reported that Mulberry leaf extract has a suppressive effect on blood glucose and increases the excretion of breath hydrogen in response to sucrose ingestion in humans [15]. Another study has determined the optimal dose of DNJ-enriched powder to suppress postprandial blood glucose [16]. However, it has not yet been reported whether processed foods containing ELM are effective in suppressing postprandial blood glucose and insulin, and in increasing the fermentation of carbohydrates in the lower intestine, thus leading to a prebiotic effect.

Collene et al. have clarified that alpha-glucosidase inhibitor containing meals increases colonic fermentation [17]. When the digestion of orally ingested carbohydrates is inhibited by Mulberry leaf extract, the unhydrolyzed carbohydrate is transferred to the large intestine, and is fermented by colonic microbes [15]. Hydrogen gas is then produced and is absorbed from the lumen in the lower intestine and excreted in the breath [18]. Therefore, breath hydrogen excretion can be used as a parameter of fermentation in the colon. We have previously reported that breath hydrogen is excreted dose-dependently and reflects the fermentability of nondigestible oligosaccharides when several dose levels of fructo-oligosaccharide and galactosyl-sucrose, which are completely fermented by colonic microbes, were ingested by human subjects [18]. Therefore, the amount of excreted breath hydrogen indicates the amount of carbohydrates that has escaped digestion in the small intestine and reached the lower intestine.

A confection that can put the inhibitory effects of ELM to immediate account is useful not only for prevention but also with regard to its therapeutic potential in type 2 diabetic patients. In our previous study, the inhibitory effects of ELM were strongest for the intestinal sucrase and next for maltase [14]. Therefore, it was necessary in the present study for the test confections to contain a sucrose-rich component. We selected test confections for which the ingredients were primarily sucrose or sucrose and starch. It is assumed that the inhibitory effects of ELM for alpha-glucosidase differed in accordance with the ratio of ELM to saccharide in the confections. Therefore, the most effective ratio of ELM to saccharides must be clarified.

The first aim of this study was to clarify the effective ratio of ELM to sucrose so as to apply this knowledge to the preparation of confections that could effectively suppress the elevation of postprandial blood glucose and insulin. The second aim was to identify the efficacy of confections prepared with the optimally effective ratio determined from the first study, using healthy human subjects. Confections such as Mizu-yokan, Daifuku-mochi, and Chiffon-cake were used to test the effects on postprandial blood glucose and insulin.

## Methods

### Subjects

Ten healthy females, aged 22.3 (SD 4.1) years with BMI 21.4 (SD 2.1) kg/m^2 ^participated. The excluded criteria were the history of diabetes, carbohydrate malabsorption, and pulmonary disease. The average and standard deviation of fasting blood glucose levels in the subjects was 86(SD 4.3) mg/100 ml. The subjects had not been treated with antibiotics or laxatives at least 2 weeks prior to the experiment. Each subject gave their informed consent to participate in the study.

### The powder of extractives from the leaves of *Morus alba*

The ELM was kindly provided by Toyotama Healthy Food Co., Ltd (Tokyo, Japan). To prepare the ELM powder, the ethanol was removed and the digestible dextrin was added to dry after the leaves were extracted with 50% ethanol. The composition of powder of the extractive from the leaves of *Morus alba *(ELM) is shown in Table 1. To extend the use of ELM, the ELM powder was made with the addition of dextrin, which makes it easy to mix with other ingredients and to dissolve in water during food-processing. The powder of ELM contains 0.77% of 1-deoxynojirimycin equivalent, containing its derivatives which inhibit alpha-glucosidase [6], and it was determined to be safety [20]. ELM has a flavor of green grasses and is khaki or dark green in color.

**Table 1:** Constituents of powder of extractives from leaves of *Morus alba*

Content	g/100 g
Water	2.2
Protein	7.2
Fat	1.2
Ash	13.8
Dextrin	73.2
Dietary fiber	1.6
1-deoxynojirimycin	0.77

Data were analyzed by Japan Food Research Laboratories.

Digestible dextrin was used.

### Test solutions and foods

Sucrose solution and three types of confection were prepared as test substances. Each type of test substance was composed of a control substance that did not contain ELM, and of ELM-containing substances with two different dosages of ELM, respectively.

#### Preparation of sucrose solutions

First, the most appropriate ratio of ELM to sucrose that markedly suppresses the postprandial elevation of blood glucose and insulin was estimated. Thirty grams of sucrose, which was used as a control solution, was dissolved in 150 ml of tap water. For the sucrose-3.0 (ratio of ELM and sucrose is 1:10) and sucrose-1.2 (that is 1:25) solutions, 3.0 g and 1.2 g of ELM were added to 30 g of sucrose solution, respectively.

#### Preparation of confections

The most effective ratio of ELM to sucrose was evaluated to be 1:10 based on the results of the first experiment using sucrose solutions; thereafter, each group of Mizu-yokan, Daifuku-mochi, or Chiffon-cake was prepared based on this ratio. Their ingredients are shown in Table 2. The constituent parts of Mizuyokan-0 (control) were 30 g of sucrose, 15 g of azuki-bean (dry), and 0.4 g of agar, and those of Daifuku-mochi-0 (control) were 30 g of sucrose, 15 g of azuki-bean, 8 g of rice-flour, and 1 g of potato-starch. The paste made from Azuki-bean can mask the flavor and color of ELM. Mizuyokan-3.0 and Daifuku-mochi-3.0 were added 3.0 g of ELM, for which the ratio to sucrose was 1:10, and Mizuyokan-1.5 and Daifuku-mochi-1.5 were added 1.5 g of ELM, with an ELM to sucrose ratio of 1:20. The ingredients of Chiffon-cake-0 (control) were applied to contain 24 g of sucrose and wheat-flour, 32 g of egg, and 4 other ingredients. As the Chiffon cake contained much more starch than Mizu-yokan and Daifuku-mochi, 6.0 g (1:4) and 3.0 g (1:8) of ELM were added to 24 g of sucrose for the Chiffon-cake-6.0 and -3.0, respectively.

**Table 2:** Ingredients of confections.

Ingredients (g/one serving)	*Mizu-yokan*			*Daifuku-mochi*			*Chiffon-cake*		
			
	0	1.5	3.0	0	1.5	3.0	0	3.0	6.0
ELM	0.0	1.5	3.0	0.0	1.5	3.0	0.0	3.0	6.0
Sucrose	30.0	30.0	30.0	30.0	30.0	30.0	24.0	24.0	24.0
Wheat-flour	-	-	-	-	-	-	24.0	24.0	24.0
Rice-flour	-	-	-	8.0	8.0	8.0	-	-	-
Potato-starch	-	-	-	1.0	1.0	1.0	-	-	-
Baking powder	-	-	-	-	-	-	0.5	0.5	0.5
Agar	0.4	0.4	0.4	-	-	-	-	-	-
Adzuki-bean*	15.0	15.0	15.0	15.0	15.0	15.0	-	-	-
Egg	-	-	-	-	-	-	32.0	32.0	32.0
Milk	-	-	-	-	-	-	4.8	4.8	4.8
Soybean-oil	-	-	-	-	-	-	5.7	5.7	5.7
Salt	-	-	-	-	-	-	0.3	0.3	0.3
Water	29.6	28.1	26.6	22.0	20.5	19.0	8.7	5.7	2.7
Total	75.0	75.0	75.0	76.0	76.0	76.0	100.0	100.0	100.0

ELM, the extractive from leaves of *Morus alba*

*Adzuki-bean was preliminary cooked to paste.

### Experimental protocol

This study was performed using a within-subject, repeated-measures design, and all subjects repeatedly ingested all of 12 kinds of test substances (3 solutions and 9 confections) with at least 1-week intervals. For first aim, all of subjects ingested 3 kinds of sucrose solution with or without ELM, and ingested 9 confections with or without ELM for second aim. Subjects ingested the test solutions within 1 min, and the confections and 150 ml of water within 5 min.

**Figure 1:**
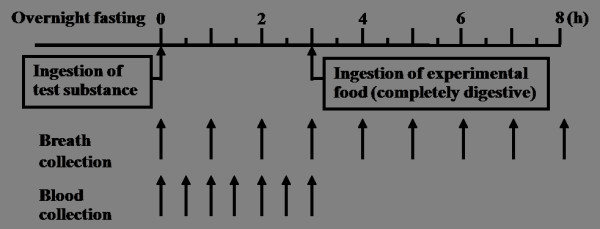
**Experimental protocol**.

The experimental protocol was carried out in accordance with our previous study [21] as shown in Figure 1. After overnight fasting of 12 h or more, the subject's health status was examined, their blood pressure and pulse rate were measured, and thereafter, a baseline blood sample and end-expiratory gas were collected. After ingestion of the test substance, blood was collected from the fingertip using a heparinized capillary tube at 30-min intervals for 3 h and 750 ml of breath after the dead-space air was eliminated were simultaneously collected at 1-h intervals for 8 h.

The subjects were prohibited from the ingestion of foods containing non-digestible carbohydrate starting at 3 days before the experimental day. During the experiment they were also prohibited from the ingestion of food and beverages except for water, sleeping, and smoking. After the final collection of blood, the subjects were given the special experimental meal, which was completely digested and breath hydrogen was not produced, to avoid a sense of starvation.

### Analysis of blood glucose, insulin, and breath hydrogen gas

Blood samples were centrifuged at 11,000 rpm (Kubota3110, Kubota Corp., Japan) for 5 min at room temperature to separate plasma. Plasma glucose and insulin concentrations were measured in duplicate by Trinder's method using glucose oxidase [22] and using an ELISA kit [23] (Seikagaku Corp., Kanagawa, Japan), respectively. Breath hydrogen concentrations were analyzed in duplicate using a simple gas chromatograph (Breath Gas Analyzer TGA2000, Teramecs Co., Ltd., Kyoto, Japan).

### Calculation and statistical analysis

The mean and standard deviation (SD) of the incremental concentrations of blood glucose and insulin from the baseline level as well as the concentrations of breath hydrogen gas were calculated using all 10 subjects; there were 9 degrees of freedom, and tested normality. Incremental areas under the curve versus time (AUCs) were calculated using the trapezoidal rule with fasting values as the baseline, and the ratio of AUCs of the test confections with added ELM was calculated in the case when the AUC of each control substance was considered to 100. One-way analysis of variance and Tukey's post hoc test were used to investigate the effects of ELM, and *p*-values less than 0.05 were considered significant with two-sided analysis using SPSS for Windows, Japan, version 11.0 (SPSS Inc., Tokyo, Japan).

### Ethics

The study protocol was approved by the ethical committees of Siebold University of Nagasaki. All of the experiments were carried out in the laboratory of Public Health Nutrition of the Graduate School of Human Health Science in Siebold University of Nagasaki.

## Results

### Effective ratio of ELM to sucrose that suppresses the increment of blood glucose and insulin

**Figure 2:**
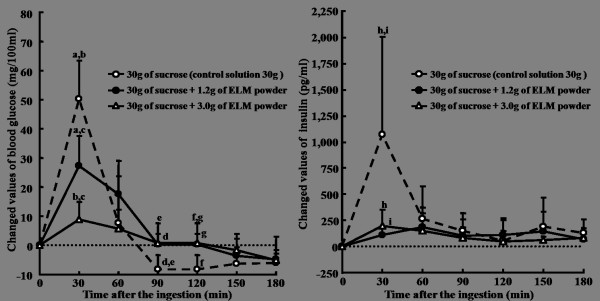
**Suppressive effects of ELM added to sucrose solutions on the increment of blood glucose and insulin in healthy subjects**. ELM: the extractive from the leaves of *Morus alba*. Data were expressed as the mean ± SD for 10 subjects in each ingestion. There was a significant difference in each same letter at each time point by ANOVA and Tukey's post hoc test, at *p *< 0.05.

The response of blood glucose and insulin after ingestion of the sucrose solution containing 3.0 g or 1.2 g of ELM is shown in Figure 2. The incremental blood glucose from the baseline was significantly increased to 50.3 mg/100 ml compared with the fasting levels at 30 min after the ingestion of 30 g of sucrose solution (*p *< 0.001); thereafter, it was attenuated to a basal level until 60 min. When 3.0 g of ELM was added to the solution containing 30 g of sucrose, the incremental blood glucose level was significantly suppressed to 8.9 mg/100 ml compared with that of the control at 30 min after the ingestion (*p *< 0.001). In the case of the addition of 1.2 g of ELM, it was significantly less than that of the control (*p *= 0.001), but the change was not as marked as that with 3.0 g of ELM (*p *= 0.008). The response of insulin after the ingestion of sucrose solution was similar to that of blood glucose, but in the cases of the addition of both 3.0 g and 1.2 g of ELM, blood insulin concentration hardly increased from the baseline level.

**Figure 3:**
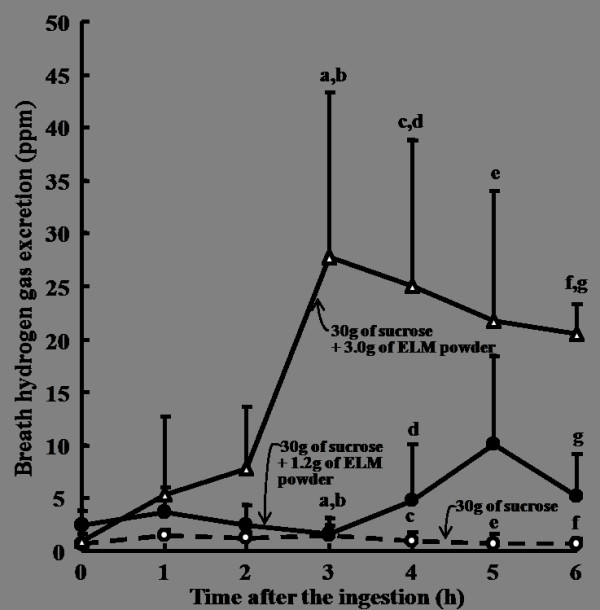
**Effects of ELM added to sucrose solutions on breath hydrogen excretion in healthy subjects**. ELM: the leaves of *Morus alba*. Data were expressed as the mean ± SD for 10 subjects in each ingestion. There was a significant difference in each same letter at each time point by ANOVA and Tukey's post hoc test, at *p *< 0.05.

The profiles of breath hydrogen excretion differed significantly between the ingestion of sucrose alone and the sucrose solutions added 3.0 g or 1.5 g of ELM, as shown in Figure 3. The breath hydrogen excretion was the greatest with the addition of 3.0 g of ELM and was negligible with the ingestion of sucrose alone. These results indicate that most of the sucrose was not digested by the inhibition of ELM to sucrase and was actively fermented by the intestinal microbes in the large intestine.

### Suppressive effects of ELM containing confections on the postprandial increment of blood glucose and insulin

**Figure 4:**
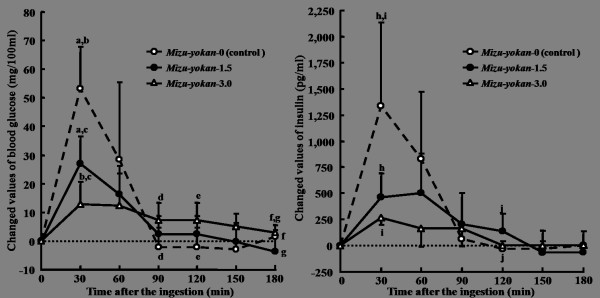
**Suppressive effects of *Mizu-yokan *added different amounts of ELM on the incremental blood glucose and insulin in healthy subjects**. ELM: the extractive from the leaves of *Morus alba*. Data were expressed as the mean ± SD for 10 subjects in each ingestion. There was a significant difference in each same letter at each time point by ANOVA and Tukey's post hoc test, at *p *< 0.05.

**Figure 5:**
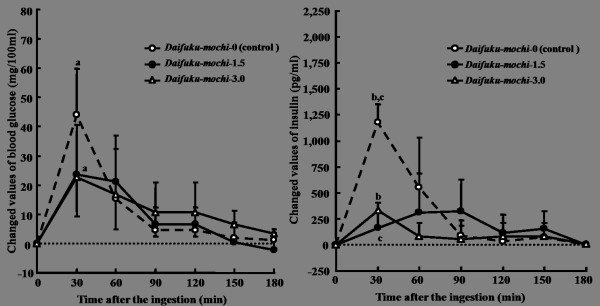
**Suppressive effects of *Daifuku-mochi *added different amounts of ELM on the incremental blood glucose and insulin in healthy subjects**. ELM: the extractive from the leaves of *Morus alba*. Data were expressed as the mean ± SD for 10 subjects in each ingestion. There was a significant difference in each same letter at each time point by ANOVA and Tukey's post hoc test, at *p *< 0.05.

**Figure 6:**
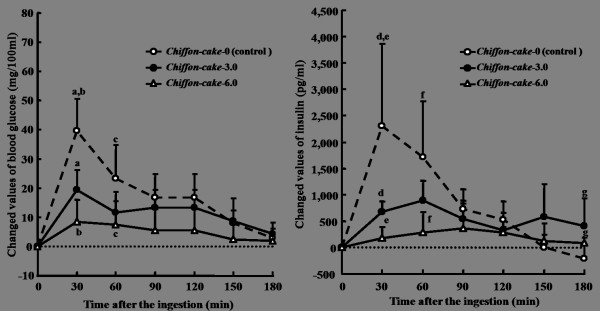
**Suppressive effects of *Chiffon-cake *added different amounts of ELM on the incremental blood glucose and insulin in healthy subjects**. ELM: the extractive from the leaves of *Morus alba*. Data were expressed as the mean ± SD for 10 subjects in each ingestion. There was a significant difference in each same letter at each time point by ANOVA and Tukey's post hoc test, at *p *< 0.05.

The incremental values of postprandial blood glucose and insulin in response to the ingestion of ELM-containing Mizu-yokan, Daifuku-mochi, and Chiffon-cake are shown in Figures 4, 5, and 6, respectively. When Mizu-yokan-3.0, to which was added 3.0 g of ELM to 30 g of sucrose, was ingested, the incremental blood glucose was significantly decreased from 53.3 to 12.8 mg/100 ml (*p *< 0.001) at 30 min after the ingestion (Figure 4). It was also significantly suppressed by the ingestion of Mizu-yokan-1.5 (*p *< 0.001), but the degree of suppression was less than that with the ingestion of Mizu-yokan-3.0 (*p *= 0.049). The responses of insulin paralleled the blood glucose response.

The postprandial increments of blood glucose were significantly suppressed by Daifuku-mochi-3.0 added 3.0 of ELM in comparison with Daifuku-mochi-0 at 30 min after the ingestion (*p *= 0.046), but it was not significant with the addition of 1.5 g of ELM (Figure 5). At 60 min after the ingestion, the blood glucose levels did not differ among Daifuku-mochi-3.0, -1.5, and -0. The response of insulin was greatly suppressed by the addition of both 3.0 and 1.5 g of ELM at 30 min after the ingestion of test substances, respectively, but it was gradually decreased until 90 min after the ingestion of Daifuku-mochi-1.5.

In the case of Chiffon-cake, the incremental blood glucose was significantly suppressed by the addition of both 3.0 g and 6.0 g of ELM at 30 min after ingestion, respectively (*p *= 0.001; *p *< 0.001), and it was dosage-dependent of ELM (Figure 6). The blood glucose levels did not return to the basal level until 150 min after the ingestion of Chiffon-cake-3.0 and -6.0. The postprandial insulin response was also suppressed at 30 min after the ingestion of both Chiffon-cake-3.0 and -6.0, however, the peak of elevation of insulin tended to be delayed by the addition of ELM.

### Comparison of the ratios of incremental AUCs of glucose and insulin until 2 h and 3 h after the ingestion of test confections

The ratios of incremental AUCs of blood glucose and insulin until 2 h and 3 h after the ingestion of ELM-containing confections versus each control are summarized in Table 3.

**Table 3:** The ratios of areas under the curve of incremental postprandial blood glucose and insulin for 2 h or 3 h after the ingestion of test confections.

	Ratio of AUC (0–2 h)				Ratio of AUC (0–3 h)			
		
Confections	Glucose		Insulin		Glucose		Insulin	
				
	Mean	SD	Mean	SD	Mean	SD	Mean	SD
*Mizu-yokan*-0	100.0^a, b^	0.0	100.0^g, h^	0.0	100.0^n, o^	0.0	100.0^s, t^	0.0
*Mizu-yokan*-1.5	59.6^a^	25.0	59.2^g, I^	35.9	59.9^n^	26.3	61.3^s, u^	36.2
*Mizu-yokan*-3.0	49.6^b^	36.3	26.7^h, I^	2.9	53.4^o^	39.1	26.7^t, u^	2.9
								
*Daifuku-mochi*-0	100.0^c, d^	0.0	100.0^j^	0.0	100.0^p, q^	0.0	100.0^v^	0.0
*Daifuku-mochi*-1.5	69.5^c^	24.7	63.3	40.4	68.5^p^	23.0	67.0	36.8
*Daifuku-mochi*-3.0	53.8^d^	17.9	34.0^j^	17.6	58.2^q^	18.1	35.7^v^	20.2
								
*Chiffon-cake*-0	100.0^e, f^	0.0	100.0^k, l^	0.0	100.0^r^	0.0	100.0^w, x^	0.0
*Chiffon-cake*-3.0	62.0^e^	23.7	51.4^k, m^	19.4	64.2	22.6	59.6^w, y^	21.7
Chiffon-cake-6.0	28.6^f^	30.2	22.0^l, m^	14.0	29.0^r^	31.7	23.6^x, y^	14.6

AUC, area under the curve; ELM, the extractive from leaves of *Morus alba*. Data is in each confection are described in experimental methods. There is a significant difference between same letters Tukey's post hoc test ANOVA (*p *< 0.05).

The incremental AUCs were varied from subject to subject but were not significantly different between 2 h and 3 h after the ingestion of each confection. The ratios of AUCs for 3 h in blood glucose were significantly decreased by the addition of both 3.0 g and 1.5 g of ELM in both Mizu-yokan (49.6 in -3.0, *p *= 0.002; 59.6 in -1.5, *p *= 0.012) and Daifuku-mochi (53.8 in -3.0, *p *= 0.001; 69.5 in 1.5, *p *= 0.015), respectively. Those in insulin were significantly decreased in Mizu-yokan, but it was not by the ingestion of Daifuku-mochi-1.5. In the case of Chiffon-cake, the ratio of AUC in blood glucose was suppressed only by the addition of 6.0 g of ELM (*p *= 0.001).

### Comparison of breath hydrogen excretion after ingestion of Chiffon-cake added 3.0 g or 6.0 g of ELM

**Figure 7:**
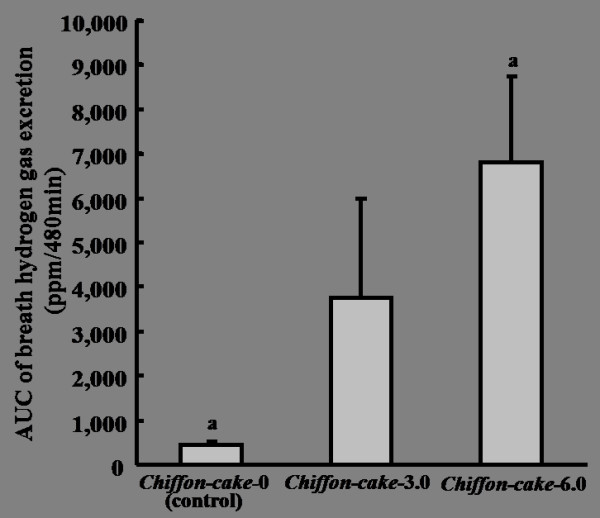
**Cumulative excretion of breath hydrogen by ingestion of ELM-containing *Chiffon-cake***. AUC: area under the curve; ELM: the extractive from the leaves of *Morus alba*. The detail constituent of *Chiffon-cake *showed in Table 2. The Data were expressed as the mean ± SD for 10 subjects. a: Cumulative excretion of breath hydrogen for 8 h after ingestion of *Chiffon-cake*-6.0 was significantly more than that of *Chiffon-cake*-0 by ANOVA and Tukey's post hoc test, at *p *< 0.05.

The excretion of breath hydrogen was determined for 8 h after the ingestion of chiffon cake, although the breath hydrogen excretion of other 2 confections was not measured (Figure 7). The breath hydrogen excretion markedly increased in chiffon cake added 6.0 g of ELM, and it was a little in chiffon cake added 3.0 g of ELM, but was more than that of chiffon cake without ELM. The excretion of breath hydrogen increased dosage-dependently of the addition of ELM (*p *= 0.002).

## Discussion

The goal of our research project was to develop functional foods with an inhibitory effect against disaccharidase to increase the quality of life of diabetic patients or to prevent diabetes mellitus. We have already clarified the inhibitory effect and mode of ELM for disaccharidase using rat and human small intestine. In addition, we have demonstrated that the elevation of postprandial blood glucose and insulin is suppressed dose-dependently when sucrose solution with ELM is orally administered to rats. In this study, we first determined the most effective ratio of ELM to carbohydrate, especially sucrose, to suppress the elevation of postprandial blood glucose and insulin, and then the suppressive efficacy of confections, which were prepared based on the effective ratio of ELM to carbohydrate, was analyzed using within-subject, repeated-measures design.

The results obtained in this study indicated that the most effective ratio of ELM to sucrose was 1 to 10, and that the suppressive effect was dose-dependent on the added ELM. The most effective ratio of ELM to sucrose (1:10) could be applied to the preparation of some kinds of confections using not only sucrose alone but also sucrose and starches. Mizu-yokan, which has a primitive composition, most effectively suppressed the increment of postprandial blood glucose and insulin. The suppressive effect of Daifuku-mochi, which contained sucrose and starch as carbohydrate, was less than that of Mizu-yokan. Chiffon-cake, which has a complicated composition, contained sucrose, starch, oil, egg, milk, and baking powder, revealed a weaker effect than Daifuku-mochi for the postprandial blood glucose and insulin. These results demonstrate that the suppressive effect of confections, which has a complicated composition contained lipid, protein and others, needs much ELM to reveal the expected effects and that the confections containing appropriate amounts of ELM produce a lowering effect on postprandial blood glucose and insulin. Furthermore, it suggests that we can effectively design confections with the expected suppressive effect.

Beverages and tablets that contain dietary fibers or polyphenols have already been developed and offered for sale with the intent of suppressing the postprandial increment of blood glucose [24-27]. These foods were designed to ingest with the other foods or meals increasing blood glucose levels. Accordingly, the lowering effect for blood glucose is not constant and is easily varied by the kind and amount of additional foods, the timing of ingestion, and other factors. However, with the confections examined in this study, it was not necessary to consider the timing of ingestion or the additional carbohydrate foods consumed in order to obtain the expected beneficial effect because the confections themselves could produce the desired lowering effect for postprandial blood glucose and insulin. Also, it is very easy to explain the benefit for the control of hyperglycemia and the advantages of such foods over other foods focused on lowering blood glucose.

The inhibitory mode of ELM is competitive with sucrase [14]. Sucrase in the small intestine is definitely inhibited by ELM ingested simultaneously, and the remaining sucrose is transferred to the large intestine, where it is spontaneously fermented by the intestinal microbes [15]. In particular, the increment of blood glucose and insulin from baseline levels and AUCs of breath hydrogen excretion are reflected by the ratio of additional ELM to sucrose. The addition of 3 g of ELM to the solution containing 30 g of sucrose indicated that most of the sucrose was not digested by the inhibition of ELM to sucrase and was actively fermented by the intestinal microbes in the large intestine. The production of breath hydrogen by the addition of ELM demonstrates that intestinal microbes utilized the sucrose which arrived at the large intestine, and it is expected that intestinal microflora was improved, thus causing prebiotic effects.

In addition, the available energy of sucrose is decreased to approximately 8.368 kJ/g (2 kcal/g) according to calculations based on the Health Promotion Act in Japan, since the retention energy is spent during fermentation [4,28]. The digestion of sucrose was found to be strongly inhibited by ELM, and the excretion of breath hydrogen was markedly observed in this study. As a result, sucrose which was not digested in the small intestine could function as a prebiotics. Accordingly, the sucrose with added one-tenth ELM brings about a suppression of postprandial elevation of blood glucose, with a reduction in available energy and prebiotic effects. In addition, these results suggest that the confections added appropriate amount of ELM can reveal beneficial effects such as a reduction of available energy and prebiotic effect.

The ratio of AUCs of both incremental blood glucose and insulin in Mizu-yokan and Daifuku-mochi were significantly lower in the addition of a one-tenth portion of ELM to sucrose compared to the absence of ELM. These results strongly support that the recommended ratio of ELM to sucrose is 1 to 10, and that a prebiotic effect can be expected with this ratio. In the case of Chiffon-cake, the results were significant only with the addition of a one-fourth portion of ELM in relation to sucrose, suggesting that the appropriate ratio of ELM increases depending on the presence of carbohydrates other than sucrose in the confection. Confections with appropriate amounts of added ELM can contribute to the control of postprandial hyperglycemia for pre-diabetic and type-2 diabetic patients. In addition, they will be able to be used for the prevention of life-style related diseases.

On the other hand, the ingestion of a sufficiently large amount of non-digestible sugar substitutes such as fructo-oligosaccharide, lactulose and maltitol causes overt osmotic diarrhea, and frequently minor symptoms such as abdominal distention and flatus, in human and animals, because non-digestible sugar substitutes, which escape digestion and absorption in the small intestine, reach the large intestine and then increase the osmotic pressure [29-31]. The carbohydrates, including sucrose, which the digestion is inhibited by added ELM are transferred to the large intestine and then increase the osmotic pressure. If large amount of confections such as Mizu-yokan or Daifuku-mochi containing appropriate amount of ELM is ingested once, osmotic diarrhea may occur. Therefore, when the confections containing appropriate amount of ELM and suppressing postprandial elevation of blood glucose are ingested by patients of diabetes mellitus and other diseases, the quantity of confection should be controlled.

## Conclusion

It was clarified that the effective ratio of ELM to sucrose, which appropriately suppressed postprandial blood glucose and insulin, was 1 to 10. Confections containing ELM that were prepared based on this ratio effectively suppressed the postprandial elevation of blood glucose and the secretion of insulin, since the digestion of sucrose and starch was certainly inhibited by the ELM. Furthermore, as they are transferred into the large intestine and fermented by intestinal microbiota, our results show that these confections could lead to an additional benefit of a prebiotic effect. The development of confections with ELM can contribute to diet therapy for type-2 diabetic patients and to the prevention of life-style related diseases.

## Competing interests

The authors declare that they have no competing interests.

## Authors' contributions

MN carried out the collection of blood and respiration, and the assay of blood glucose and insulin, and breath hydrogen. SN carried out the management of subjects, performed the statistical analysis, and helped to draft the manuscript. TO conceived of the study, and participated in its design and coordination. All authors read and approved the final manuscript.
